# Monitoring the parasite load in chronic Chagas disease patients: comparison between blood culture and quantitative real time PCR

**DOI:** 10.1371/journal.pone.0208133

**Published:** 2018-11-29

**Authors:** Daniella Alchaar D’Ávila, Lúcia Maria C. Galvão, Giovane R. Sousa, Constança Britto, Otacilio C. Moreira, Egler Chiari

**Affiliations:** 1 Programa de Pós-Graduação em Parasitologia, Departamento de Parasitologia, Instituto de Ciências Biológicas, Universidade Federal de Minas Gerais, Belo Horizonte, MG, Brazil; 2 Section on Immunobiology, Joslin Diabetes Center, Harvard Medical School, Boston, MA, United States of America; 3 Laboratório de Biologia Molecular e Doenças Endêmicas, Instituto Oswaldo Cruz, FIOCRUZ, RJ, Brazil; Universidad de Chile, CHILE

## Abstract

**Background:**

Despite the improvements in diagnostic tools for detection of *Trypanosoma cruzi* in human blood samples, the isolation of parasite from bloodstream in the chronic phase of Chagas disease is challenging. Thus, there is an increasing interest in the development of strategies that allow an accurate monitoring of the parasite load in bloodstream of Chagas disease patients. Given that, the comparison of a classical diagnostic method such as blood culture and multiplex quantitative real-time PCR (qPCR) was few explored so far. Therefore, this study aimed to compare the detection and quantification of *T*. *cruzi* load in the circulating blood of patients with chronic Chagas disease, using blood culture and qPCR techniques.

**Methods⁄Principal findings:**

The multiplex real-time quantitative PCR assay (qPCR) based on *TaqMan* technology was evaluated in 135 blood samples from 91 patients with chronic Chagas disease presenting indeterminate (asymptomatic, n = 23) and cardiac (chronic cardiomyopathy, n = 68) forms, in comparison with the classical blood culture (BC) technique. The total positivity of qPCR and BC was 58.5% and 49.6%, respectively. The median parasite load of all positive patients was 1.18 [0.39–4.23] par. eq.⁄mL, ranging from 0.01 to 116.10 par. eq.⁄mL. We did not find significant differences between *T*. *cruzi* load with age and distinct clinical manifestations of patients.

**Conclusions/Significance:**

Our data suggest that qPCR can be an auxiliary tool for studies that require *T*. *cruzi* isolation from the bloodstream of patients with chronic Chagas disease, after the establishment of a parasite load cut-off that guarantees a relative success rate of parasite isolation using BC technique.

## Introduction

The diagnosis of *Trypanosoma cruzi* infection should be carried out using different methodologies, depending on the stage of the disease. In the acute phase of the infection, the parasitemia is high in the peripheral circulation and the diagnosis of Chagas disease can be performed by direct examination of the blood. In contrast, in the chronic phase of the disease the parasitemia is subpatent, transient and depends on the immune response of each patient [1]. Current methods available for parasitological and serological diagnosis have limitations in sensitivity and specificity, especially when applied for the diagnosis in chronic phase of the disease. The major limitation of serological methods is the low specificity, due to cross-reactions with other trypanosomatids present in endemic areas such as *Leishmania* sp. and *Trypanosoma rangeli* [2–4]. Another difficulty is that following anti-*T*. *cruzi* specific treatment, serological methods remain positive for several years. In the chronic phase of the disease, the post treatment serological reversion is less than 10%, which impairs the efficacy of therapeutic evaluation [5–10]. Indirect parasitological diagnostic methods such as xenodiagnosis and blood culture (BC) depend on the presence of at least one intact trypomastigote form for its growth in culture medium. The results of these methods can take up to 120 days and still be doubtful [6, 11–13]. Negative results of BC and/or xenodiagnosis may be due to low parasitemia observed in the chronic phase of Chagas disease and do not rules out the possibility of infection. On the other hand, a positive test has an absolute diagnostic value [14]. In individuals with inconclusive serology, BC is an important tool for identifying *T*. *cruzi*, and when positive it is possible the parasite isolation for biological, biochemical and molecular studies [15,16].

The main technique that has been tested for the research of *T*. *cruzi* directly in the blood of chronic-affected patients is the conventional PCR, based on the use of synthetic oligonucleotides that amplify specific DNA sequences of the parasite, presenting high sensitivity and promising results, although it is not feasible for a quantitative evaluation [17–20]. The difficulties in the diagnosis of Chagas disease in chronic phase justify the interest and the necessity of implementation of a direct and more sensitive method that allows monitoring the presence of the parasite and confirming the etiology of the disease.

In the last decade, the methodology used for the detection of genes and specific sequences of *T*. *cruzi* has been improved with the development of different real-time PCR systems. An automated quantitative approach based on the use of fluorogenic probes (TaqMan) or fluorescent dyes with DNA affinity (SYBRGreen) has been useful for demonstrating the absolute levels of *T*. *cruzi* circulating in infected individuals. This methodology represents a major advance in molecular diagnostic methods and gives support to research laboratories, particularly facilitating the quantification of DNA or RNA fragments in different biological samples and, capable of accurately estimates *T*. *cruzi* parasite load of patients in chronic phase of Chagas disease [21–34]. In addition, it allows the monitoring of disease progression, evaluation of parasitemia in response to specific treatment, congenital infection and early detection of reactivation [21–34].The qPCR is a more advantageous methodology when compared to conventional PCR and BC, since it presents higher sensitivity and early outcome for confirming the infection. Furthermore, it evaluates and quantifies the parasite load, being useful for medical decision regarding the introduction or not of specific therapeutic against *T*. *cruzi* infection. The aim of this study was to evaluate the qPCR (TaqMan system) and blood culture strategies for detecting *T*. *cruzi* load in asymptomatic and cardiac patients with chronic Chagas disease without previous etiological treatment, since the comparison of classical parasitological method BC with qPCR was few explored. Genotyping was performed to determine the genetic profile of *T*. *cruzi* in newly isolated strains of infected patients.

## Materials and methods

### Study population

This study included 91 patients in the chronic phase of Chagas disease from different endemic regions of the state of Minas Gerais (Southern Brazil). All patients were adults and had at least two positive conventional serological tests for *T*. *cruzi* and were selected at the Referral Outpatient Center for Chagas Disease at the Clinical Hospital of the *Universidade Federal de Minas Gerais* (UFMG). Patients were subjected to a standard screening protocol that included medical history, physical examination, ECG, laboratory and chest X-ray examinations, disease evolution by echocardiography and characterization according to the clinical classification of chronic Chagas disease [35]. None of the patients were undergoing etiological treatment nor had been previously treated for *T*. *cruzi* infection. Blood samples for BC (30 mL) and qPCR (5 mL) were collected at the same time for each patient. Amongst 91 patients, 44 subjects (48%) had two blood samples collected prospectively within a range interval of two and three years, aiming to evaluate the parasitemia over time in patients with chronic Chagas disease, with a total of 135 samples. This study comprises patients from a broad project on the clinical, parasitological, molecular and immunological studies that has been developed in our laboratory since 2011.

### Ethical statement

The study was approved by the Research Ethic Committee of the *Universidade Federal de Minas Gerais* (protocol COEP-ETHIC 0559.0.203.000-11/2012/UFMG), and all participants provided written informed consent.

### Blood culture

Blood culture (BC) was performed with 30 mL of venous blood collected in heparinized vacuum tubes and red cells were recovered from the plasma by centrifugation at 300 × *g* for 10 min at 4°C [12]. The packed red blood cells were washed once and re-suspended in 6 mL of LIT (Liver Infusion Tryptose), mixed and distributed into six plastic tubes (Falcon, USA) containing 3 mL of LIT. The plasma supernatant was centrifuged at 900 × g for 20 min at 4°C, and 5 mL LIT was added to the precipitated cells. All tubes were maintained at 28°C, mixed gently twice a week, and examined monthly for up to 120 days. Microscopic examination was carried out in 10 μL aliquots of each preparation under a 22-mm^2^coverslip at a magnification of 400×.

### Genotyping of *Trypanosoma cruzi* isolates

*T*. *cruzi* was isolated from all clinical samples with positive BC and the genotyping was performed by conventional PCR and RFLP, using three different parasite molecular targets: D7 domain 24Sα ribosomal (rRNA) gene [36], mitochondrial cytochrome oxidase subunit 2 gene (COII) [37], and the intergenic region of spliced leader genes [38], as markers for the six discrete typing units (DTUs) [39]. The following, TcI (Col1.7G2 Colombiana clone), TcII (JG), TcIII (222), TcIV (CAN III clone), TcV (3253 Lages-Silva et al.: unpublished data), and TcVI (CL) were used as reference strains and DTU controls [37,39].

### DNA processing for absolute quantification by qPCR assays

For each patient, five milliliters of venous blood were collected and immediately mixed with an equal volume of 6M Guanidine Hydrochloride / 0.2 M ethylenediaminetetraacetic acid buffer (EDTA) solution, pH 8.0. The Guanidine-EDTA Blood lysates (**GEB**) were boiled during 15 min and stored at 4°C, as previously described [40]. Extraction of DNA was processed from 300 μL **GEB** using the High Pure PCR Template Preparation kit, according to the instruction provided by the manufacturer (Roche Diagnostics Corp., Indiana, USA). A linearized p-Zero plasmid containing a sequence of *Arabidopsis thaliana* was used as exogenous internal reference (Internal Amplification Control, IAC) [23,25]. Each round of DNA extraction was performed using 12 blood samples, being 11 of patients and 1 of a negative control (**GEB**^-^**)** for the DNA extraction. After extraction, DNA was stored at -20° C until the time of use in qPCR.

### Standard curves and positive controls

For the construction of the standard curve and the generation of positive controls used in qPCR, **GEB**^**-**^ from healthy individuals were spiked with 10^6^ epimastigote forms/mL of *T*. *cruzi*, Y strain (spiked **GEB**^**+**^). This strain corresponds to the discrete typing unit (DTU) II and was selected due to the high prevalence of this DTU and its association with human infection in the State of Minas Gerais/MG [16,41,42]. Total DNA was purified as previously described, followed by serial dilutions to obtain the concentrations of 10^4^, 10^3^, 10^2^, 10^1^, 10^0^ and 0.5 par. eq./mL. As diluent, DNA extracted from blood sample of a healthy individual (**GEB**^**-**^) was used. Each dilution was correlated to one point of the standard curve for the absolute quantification of parasite load in the clinical samples. DNA extracted from **GEB**^**+**^ spiked with *T*. *cruzi* to reach the concentrations of 10^2^ and 10^0^ par. eq./mL were also used as positive controls for the qPCR, in each assay.

### Absolute quantification by qPCR assays

The qPCR was performed according to a methodology previously proposed [25], using the multiplex *TaqMan* system targeting the *T*. *cruzi* nuclear satellite DNA and IAC. The qPCR reactions were carried out with 5 μL of DNA, using FastStart Universal Probe Master Mix (*Roche Diagnostics GmbHCorp*, *Mannheim*, *Germany*) in a final volume of 20 μL. The amplifications were carried out in the Step One Plus Real-Time PCR system (Applied Biosystems, USA) using 750 nM of Cruzi 1 and Cruzi 2 primers, 50 nM of Cruzi 3 probe, 100nM of IAC Fw and IAC Rv primers and 50 nM IAC Tq probe. The oligonucleotide sequences were: Cruzi 1 (ASTCGGCTGATCGTTTTCGA), Cruzi 2 (AATTCCTCCAAGCAGCGGATA) and Cruzi 3 probe (FAM-CACACACTGGACACCA-NFQ-MGB), IAC Fw (ACCGTCATGGAACAGCACGTA), IAC Rv (CTCCCGCAACAAACCCTATAAAT) and IAC Tq probe (VIC-AGCATCTGTTCTTGAAGGT-NFQ-MGB) [25]. PCR cycling conditions were: 95°C for 10 min, followed by 40 cycles at 95°C 15s and 58°C for 1min. To analyze the results, the threshold was set at 0.02. Clinical samples were tested in duplicate, and considered positive when the fluorescent signal of both technical replicates cross the threshold or negative when the fluorescent signal of both technical replicates did not cross the threshold.

### Statistical analysis

Pearson’s correlation was used to verify the linear relationship between the parasite load of *T*. *cruzi* (par. eq./mL) detected in the clinical samples (qPCR), patient age and number of positive tubes in BC. Mann-Whitney-Wilcoxon and Kruskal-Wallis tests [43] were used, respectively, for comparison of *T*. *cruzi* parasite load (par. eq./mL) with the cardiac clinical form of patients and the different levels of heart disease. Pearson chi-squared test was used to compare the positivity of BC and qPCR in the clinical samples of patients with two blood collections (samples 1 and 2). Kappa coefficient concordance and 95% confidence intervals were used to quantify the degree of agreement between the results of BCs and qPCR [44,45] in clinical samples of patients with two blood collections. To confirm or refute the evidence found by the tests mentioned above, a 5% significance level was used.

## Results

### Characteristics of the study population

Overall, 25.3% (23/91) were patients with the chronic indeterminate form of Chagas disease and 74.7% (68/91) showed different degrees of cardiac involvement. Among the patients with the indeterminate form of Chagas disease, 34.8% (8/23) were male, with ages ranging from 33 to 70 years (mean of 44±10.3 years). Amongst patients with chronic Chagas cardiomyopathy, 66.2% (45/68) of were male, with ages ranging from 25 to 81 years (mean of 54±10.3 years).

### Detection of *T*. *cruzi* by blood culture

Sixty-seven (49.6%) of the 135 clinical samples of patients with chronic Chagas disease presented positive BCs. Data concerning blood collection date, age, *T*. *cruzi* DTU, BC positivity, parasite load, clinical form of disease for each patient are given in Tables 1 and 2. A total of 63 *T*. *cruzi* isolates was obtained by positive blood culture. Of these, sixty-one are associate with discrete typing unit (DTU) II and two isolates from patients with cardiac and indeterminate form of the disease, respectively, were classified as DTU III or IV and DTU V or VI (Tables 1 and 2).

**Table 1:** Comparison of blood culture, *T*. *cruzi* genotype and parasite load in asymptomatic patients with chronic Chagas disease.

Blood Sample	Collection Date	Number of positive tubes	Blood culture	Parasite load ± SD (par. eq.⁄mL)	DTU	Age
009a	09⁄23⁄2011	1	POS	0.79**±**0.18	TcII	58
0020a	10⁄21⁄2011	0	NEG	NEG	-	48
0020b	09⁄19⁄2014	2	POS	0.37**±**0.26	TcII	
0024a	10⁄25⁄2011	6	POS	36.82**±**3.50	TcII	33
0024b	11⁄12⁄2014	5	POS	13.25**±**3.03	TcII	
0025a	01⁄11⁄2011	0	NEG	NEG	-	40
0040a	11⁄18⁄2011	1	POS	NEG	TcII	62
0041a	11⁄18⁄2011	0	NEG	NEG	-	56
0041b	09⁄26⁄2014	0	NEG	0.09**±**0.06	-	
0048a	11⁄29⁄2011	7	POS	85.81**±**6.22	TcII	38
0052a	12⁄02⁄2011	1	POS	NEG	ND	37
0054a	12⁄06⁄2011	1	POS	NEG	TcV or VI	38
0054b	11⁄14⁄2014	0	NEG	NEG	-	
0061a	02⁄13⁄2012	0	NEG	2.97**±**0.02	-	42
0061b	10⁄03⁄2014	0	NEG	0.32**±**0.08	-	
0062a	02⁄13⁄2012	0	NEG	NEG	-	36
0062b	10⁄03⁄2014	0	NEG	0.04**±**0.01	-	
0063a	03⁄02⁄2012	0	NEG	0.01**±**0.01	-	44
0063b	09⁄19⁄2014	0	NEG	0.30**±**0.16	-	
0064a	03⁄02⁄2012	1	POS	NEG	TcII	52
0064b	11⁄28⁄2014	2	POS	3.15**±**0.43	TcII	
0067a	03⁄09⁄2012	2	POS	NEG	TcII	70
0069a	03⁄20⁄2012	0	NEG	2.58**±**1.42	-	37
0069b	10⁄03⁄2014	2	POS	0.10**±**0.03	TcII	
0072a	03⁄27⁄2012	0	NEG	4.81**±**0.77	-	52
0076a	04⁄03⁄2012	0	NEG	NEG	-	36
0078a	04⁄17⁄2012	0	NEG	NEG	-	60
0078b	09⁄12⁄2014	0	NEG	0.27**±**0.10	-	
0084a	04⁄24⁄2012	0	NEG	0.55**±**0.15	-	37
0086a	04⁄24⁄2012	0	NEG	NEG	-	37
0086b	09⁄26⁄2014	2	POS	0.17**±**0.03	ND	
0088a	04⁄05⁄2012	3	POS	0.96**±**0.34	TcII	34
0092a	05⁄15⁄2012	0	NEG	NEG	-	43
0092b	11⁄18⁄2014	0	NEG	NEG	-	
0094a	05⁄29⁄2012	0	NEG	0.31**±**0.02	-	36

SD: standard deviation, par. eq./mL: parasite equivalent per milliliter of blood, POS: positive, NEG: negative, ND: not done, a: first sample collected from the patient, b: second sample collected from the patient, DTU: discrete typing units.

**Table 2:** Comparison of blood culture, *T*. *cruzi* genotype and parasite load in patients with different degrees of chronic Chagas disease cardiomyopathy.

Blood sample	Collection date	CCC	Number of positive tubes	Blood Culture	Parasite load ± SD (par. eq.⁄mL)	DTU	Age
001a	09⁄09⁄2011	CCC5	7	POS	116.10 ±4.37	TcII	59
002a	09⁄09⁄2011	CCC3	5	POS	51.74 ±16.63	TcII	41
003a	09⁄13⁄2011	CCC4	2	POS	2.01±0.76	TcII	56
004a	09⁄13⁄2011	CCC3	1	POS	1.07±0.44	TcII	51
005a	09⁄13⁄2011	CCC5	5	POS	20.86±4.71	TcII	71
006a	09⁄13⁄2011	CCC3	0	NEG	NEG	-	67
006b	09⁄26⁄2014	CCC3	0	NEG	0.05±0.02	-	
007a	09⁄23⁄2011	CCC5	1	POS	3.07±0.56	ND	45
008a	09⁄23⁄2011	CCC5	3	POS	0.41±0.35	TcII	55
0010a	09⁄27⁄2011	CCC5	3	POS	4.71±0.06	TcII	60
0012a	09⁄27⁄2011	CCC4	0	NEG	NEG	-	64
0012b	11⁄21⁄2014	CCC4	0	NEG	NEG	-	
0013a	10⁄04⁄2011	CCC3	4	POS	0.47±0.27	TcII	48
0014a	10⁄04⁄2011	CCC3	0	NEG	NEG	-	81
0015a	10⁄04⁄2011	CCC3	0	NEG	0.41±0.07	-	34
0015b	11⁄21⁄2014	CCC3	0	NEG	2.65±1.88	-	
0016a	10⁄21⁄2011	CCC4	1	POS	0.29±0.18	TcII	46
0016b	09⁄19⁄2014	CCC4	0	NEG	0.06±0.02	-	
0017a	10⁄21⁄2011	CCC1	5	POS	24.51±3.23	TcII	58
0017b	11⁄12⁄2014	CCC1	4	POS	3.74±2.78	TcII	
0018a	10⁄21⁄2011	CCC2	5	POS	12.79±1.49	TcII	46
0018b	09⁄12⁄2014	CCC2	1	POS	0.75±0.54	TcII	
0019a	10⁄21⁄2011	CCC3	5	POS	36.09±4.53	TcII	58
0019b	11⁄14⁄2014	CCC3	7	POS	5.34±2.22	TcII	
0021a	10⁄25⁄2011	CCC2	2	POS	0.41±0.35	TcII	50
0022a	10⁄25⁄2011	CCC3	1	POS	14.94±2.86	ND	58
0023a	10⁄25⁄2011	CCC5	5	POS	2.52±0.022	TcII	70
0023b	11⁄14⁄2014	CCC5	1	POS	NEG	TcII	
0026a	11⁄01⁄2011	CCC1	4	POS	0.69±0.20	TcII	25
0027a	11⁄01⁄2011	CCC5	0	NEG	NEG	-	56
0027b	08⁄29⁄2014	CCC5	0	NEG	NEG	-	
0028a	11⁄04⁄2011	CCC5	0	NEG	NEG	-	57
0029a	11⁄04⁄2011	CCC5	0	NEG	NEG	-	59
0030a	11⁄04⁄2011	CCC5	0	NEG	NEG	-	52
0031a	11⁄04⁄2011	CCC5	2	POS	4.75±0.11	TcII	59
0032a	11⁄07⁄2011	CCC3	4	POS	9.39±6.24	TcII	49
0033a	11⁄07⁄2011	CCC1	0	NEG	NEG	-	67
0033b	09⁄26⁄2014	CCC1	0	NEG	0.39±0.25	-	
0034a	11⁄07⁄2011	CCC5	5	POS	2.79±0.79	TcII	48
0035a	11⁄07⁄2011	CCC5	1	POS	NEG	TcII	68
0036a	11⁄07⁄2011	CCC3	0	NEG	NEG	-	77
0037a	11⁄07⁄2011	CCC4	4	POS	1.47±0.02	TcII	38
0038a	11⁄07⁄2011	CCC5	5	POS	1.46±0.76	TcII	52
0038b	12⁄05⁄2014	CCC5	0	NEG	2.06±1.07	-	
0039a	11⁄18⁄2011	CCC3	0	NEG	NEG	-	44
0039b	11⁄12⁄2014	CCC3	0	NEG	NEG	-	
0042a	11⁄18⁄2011	CCC5	1	POS	NEG	Tc II	55
0043a	11⁄22⁄2011	CCC5	0	NEG	NEG	-	58
0043b	09⁄26⁄2014	CCC5	0	NEG	0.07±0.01	-	
0044a	11⁄22⁄2011	CCC5	2	POS	0.91±0.38	TcII	66
0044b	11⁄14⁄2014	CCC5	2	POS	0.72±0.51	TcII	
0046a	11⁄29⁄2011	CCC5	4	POS	1.86±0.63	TcII	61
0047a	11⁄29⁄2011	CCC3	2	POS	NEG	TcII	60
0049a	12⁄02⁄2011	CCC5	2	POS	1.18±0.23	TcII	59
0049b	12⁄05⁄2014	CCC5	0	NEG	NEG	-	
0050a	12⁄02⁄2011	CCC5	1	POS	1.71 ±1.02	TcIII or IV	58
0050b	11⁄14⁄2014	CCC5	0	NEG	NEG	-	
0053a	12⁄06⁄2011	CCC5	0	NEG	NEG	-	55
0053b	11⁄18⁄2014	CCC5	1	POS	NEG	TcII	
0055a	12⁄06⁄2011	CCC5	0	NEG	NEG	-	69
0055b	10⁄03⁄2014	CCC5	0	NEG	0.94±0.17	-	
0056a	12⁄06⁄2011	CCC5	2	POS	4.98±2.12	TcII	38
0056b	11⁄28⁄2014	CCC5	0	NEG	1.17±0.52	-	
0057a	12⁄06⁄2011	CCC5	2	POS	3.57±2.99	TcII	36
0058a	02⁄10⁄2012	CCC5	0	NEG	NEG	-	58
0059a	02⁄10⁄2012	CCC4	1	POS	NEG	TcII	41
0059b	11⁄28⁄2014	CCC4	1	POS	NEG	TcII	
0060a	02⁄10⁄2012	CCC5	0	NEG	33.30±0.05	-	53
0065a	03⁄06⁄2012	CCC4	5	POS	NEG	Tc II	53
0066a	03⁄06⁄2012	CCC5	0	NEG	17.31±1.82	-	57
0066b	29⁄08⁄2014	CCC5	0	NEG	0.22±0.07	-	
0068a	03⁄20⁄2012	CCC5	2	POS	NEG	TcII	67
0068b	12⁄05⁄2014	CCC5	4	POS	22.33±0.01	TcII	
0070a	03⁄23⁄2012	CCC5	0	NEG	NEG	-	56
0070b	10⁄03⁄2014	CCC5	0	NEG	0.24±0.17	-	
0071a	03⁄27⁄2012	CCC5	0	NEG	NEG	-	44
0071b	11⁄14⁄2014	CCC5	0	NEG	NEG	-	
0073a	03⁄27⁄2012	CCC5	2	POS	13.89±4.46	TcII	54
0074a	03⁄27⁄2012	CCC5	1	POS	2.04±1.11	TcII	54
0075a	03⁄27⁄2012	CCC5	0	NEG	NEG	-	56
0075b	09⁄26⁄2014	CCC5	0	NEG	0.24±0.06	-	
0077a	04⁄03⁄2012	CCC5	0	NEG	NEG	-	59
0079a	04⁄17⁄2012	CCC3	0	NEG	NEG	-	53
0079b	09⁄12⁄2014	CCC3	1	POS	0.68±0.61	TcII	
0080a	04⁄17⁄2012	CCC2	1	POS	1.98±1.01	TcII	55
0081a	04⁄20⁄2012	CCC5	0	NEG	NEG	-	66
0081b	08⁄29⁄2014	CCC5	0	NEG	NEG	-	
0083a	04⁄20⁄2012	CCC3	0	NEG	NEG	-	35
0083b	11⁄21⁄2014	CCC3	2	POS	2.59±0.98	TcII	
0087a	04⁄24⁄2012	CCC5	1	POS	0.78±0.56	TcII	35
0089a	05⁄08⁄2012	CCC1	4	POS	3.65±1.18	TcII	53
0089b	09⁄12⁄2014	CCC1	2	POS	NEG	TcII	
0090a	05⁄15⁄2012	CCC5	0	NEG	NEG	-	54
0090b	09⁄12⁄2014	CCC5	1	POS	1.74±0.36	TcII	
0091a	05⁄15⁄2012	CCC3	0	NEG	NEG	-	59
0091b	11⁄18⁄2014	CCC3	0	NEG	NEG	-	
0093a	05⁄15⁄2012	CCC5	1	POS	0.99±0.27	TcII	58
0093b	09⁄05⁄2014	CCC5	0	NEG	0.07±0.04	-	
0095a	05⁄29⁄2012	CCC4	3	POS	0.51±0.38	TcII	46
0096a	05⁄29⁄2012	CCC2	0	NEG	0.09±0.02	-	49

CCC1 to 5: chronic Chagas cardiomyopathy in different degrees of cardiac involvement, SD: standard deviation, par. eq./mL: parasite equivalent per milliliter of blood, POS: positive, NEG: negative, ND: not done, a: first sample of the patient, b: second sample of the patient, DTU: discrete typing units.

Among the 44 patients with two collected blood samples, 22.7% (10⁄44) showed positive BC in the two blood harvesting and 45.5% (20/44) showed negative BC in both samples. On the other hand, 15.9% (7/44) presented positive BC in the first and negative in the second sample. The same amount of samples 15.9% (7⁄44) presented negative BC in the first and positive in the second sample (Table 3).The analysis of BC results showed that the positivity observed in the first and second samples were the same [38.64% (17⁄44)], statistically demonstrating an equality in the first and second sample (p-value = 0.500) (Table 3).

**Table 3:** Percentage of concordance, point and interval estimates of kappa coefficient according to qPCR and blood culture methods in 44 Chagas disease patients with two blood harvesting.

Method	1st sample	2nd sample	Percentage(Number of patients/total)	Agreement Coefficient	Type of agreement
**qPCR**	Positive	Positive	31.8 (14/44)	0.083 [-0.212; 0.379]	Slight
	Positive	Negative	9.1 (4/44)		
	Negative	Positive	36.4 (16/44)		
	Negative	Negative	22.7 (10/44)		
**Blood** **Culture**	Positive	Positive	22.7 (10/44)	0.329 [0.034; 0.624]	Fair
	Positive	Negative	15.9 (7/44)		
	Negative	Positive	15.9 (7/44)		
	Negative	Negative	45.5 (20/44)		

### *Trypanosoma cruzi* parasite load in chronic Chagas disease patients

**Fig 1:**
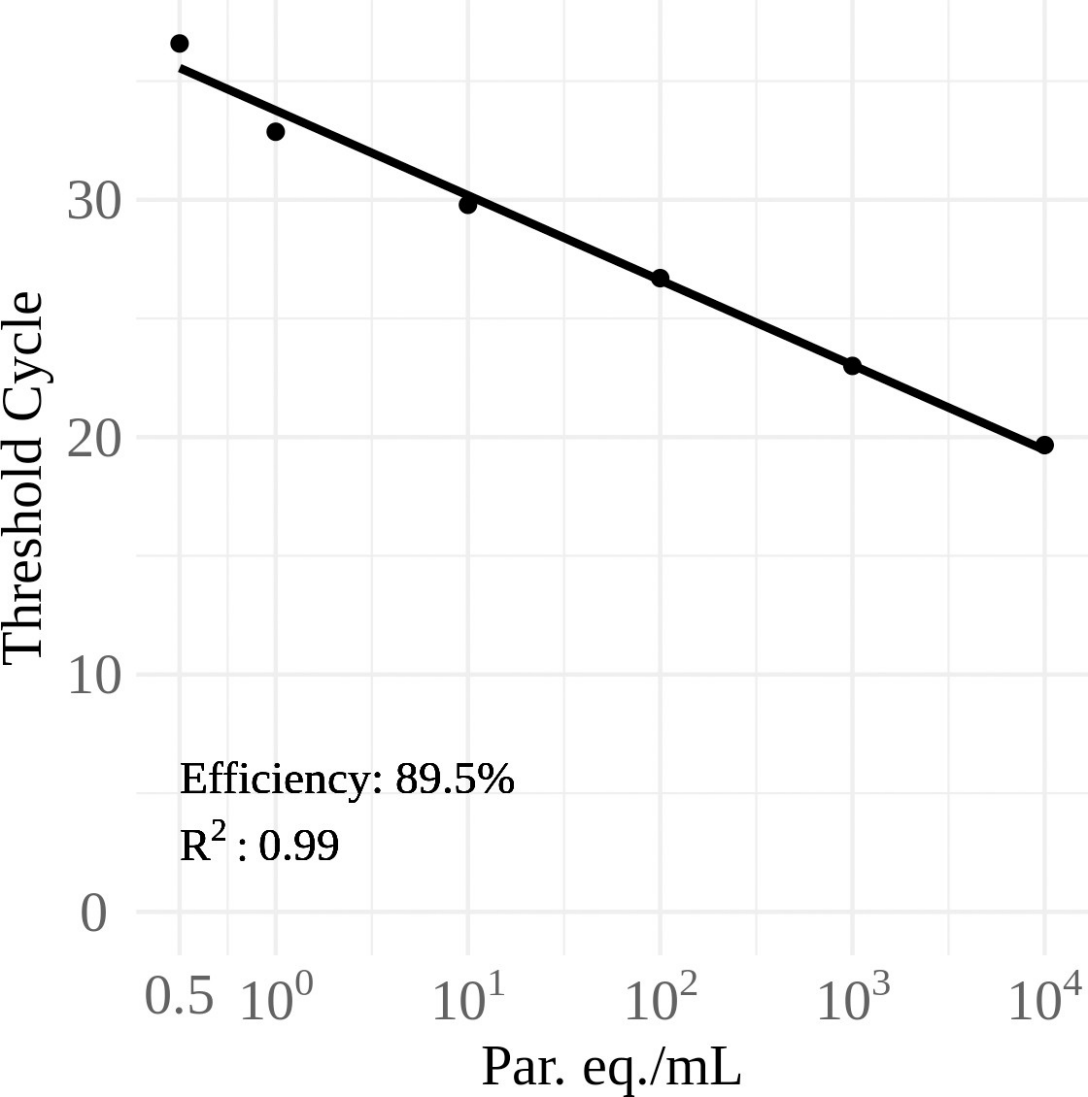
Dynamic range for *Trypanosoma cruzi* quantification by Real Time qPCR. TaqMan qPCR was carried out with serial diluted DNA extracted from blood spiked with *T*. *cruzi* [Y strain], ranging from 10^4^ to 0.5 par. eq.⁄mL (parasite equivalent per milliliter of blood).

Parasite loads were determined by qPCR absolute quantification in a TaqMan multiplex assay targeting *T*. *cruzi* satellite DNA and the internal control, IAC. It was possible to observe the dynamic range from 10^4^ to 0.5 parasite equivalents /mL, as previously reported [25,28], with efficiency of 89.5% and coefficient of linearity (r^2^) of 0.99 (Fig 1).

**Fig 2:**
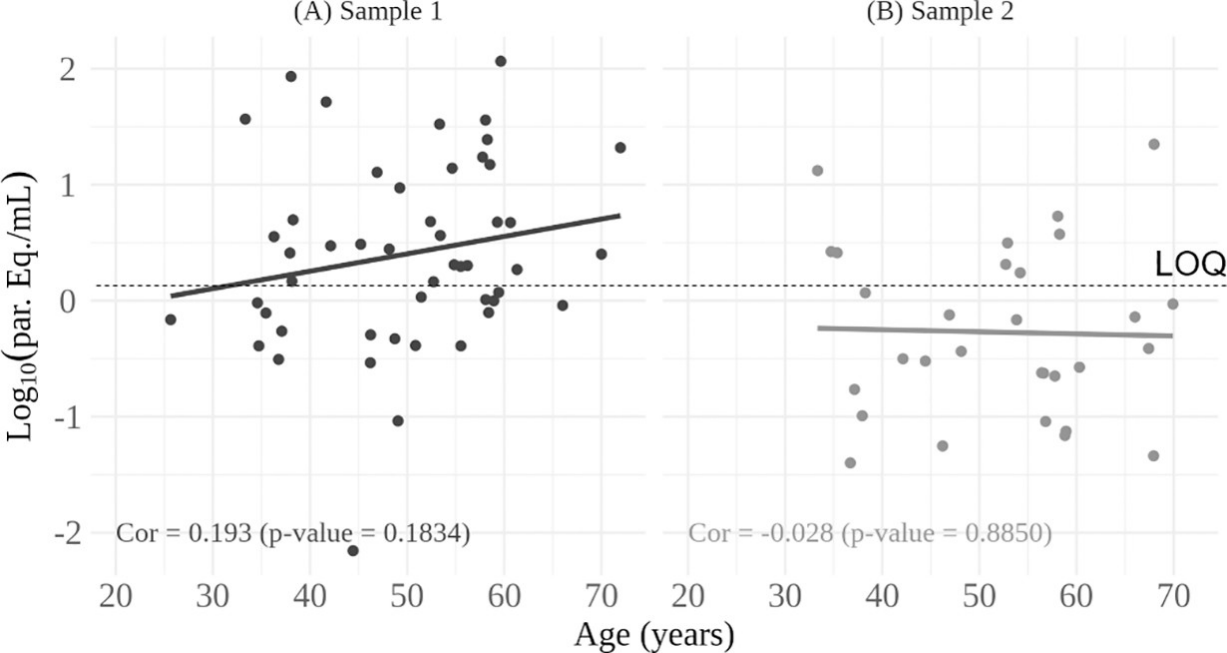
Relationship between parasite load and age of patients with chronic Chagas disease. The number of positive qPCR results for the first and second clinical samples was respectively, 49 **(A)** and 30 **(B)**. LOQ: Limit of Quantification [25].

**Fig 3:**
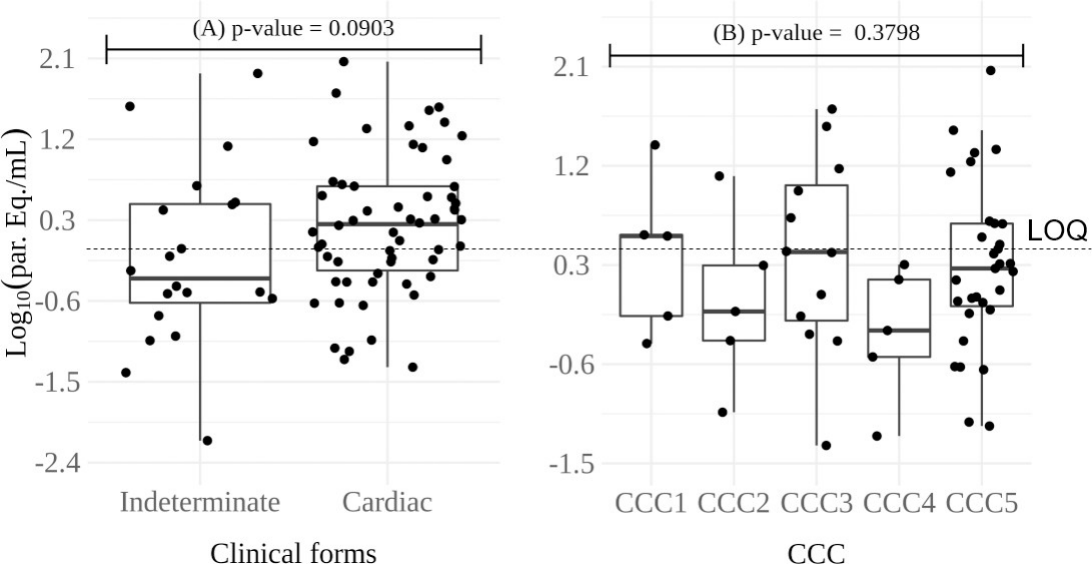
Boxplots of parasite load of chronic Chagas disease patients. (A) Patients with clinical forms, indeterminate or cardiac, and p-value of the Mann-Whitney-Wilcoxon test. (B) Patients presenting chronic Chagas’ cardiomyopathy (CCC to 5) in different degrees of cardiac involvement and p-value of the Kruskal-Wallis test. Par. eq./mL: parasite equivalent per milliliter of blood, CCC1 to 5: different degrees of cardiac involvement. LOQ: Limit of Quantification [25].

Total qPCR positivity in clinical samples was 58.5% (79/135). Data on collection date, age, DTU, parasite loads and clinical form of the disease for each patient can be seen in Tables 1 and 2.The median parasite load of all positive samples was 1.18 par. eq.⁄mL, varying between 0.01 and 116.10 par. eq.⁄mL. The median parasite load of patients with indeterminate clinical form was 0.46 [0.24–3.02] par. eq.⁄mL, varying between 0.01 and 85.81 par. eq.⁄mL, and 1.74 [0.60–4.74] par. eq.⁄mL for the cardiac patients ranging from 0.05 to 116.10 par. eq.⁄mL (Tables 1 and 2). Analyzing the data from Figs 2 and 3, we found no correlation between *T*. *cruzi* loads and the age or clinical manifestation of the disease.

Clinical samples of 44 patients with two blood harvesting were evaluated and compared, and presented parasite loads with approximated values. Only clinical samples from patients 17, 18, 19, 24 and 66 showed differences in parasite load when the second sample was evaluated (Tables 1 and 2). The qPCR was positive in 31.8% (14/44) samples from patients with two blood harvesting and 22.7% (10/44) were negative in both samples. We observed that 9.1% (4/44) presented positive qPCR in the first and were negative in the second sample. In contrast, 36.4% (16/44) presented negative qPCR in the first and positive in the second sample (Table 3). The qPCR positivity increased from 40.9% (18/44) to 68.2% (30/44) (p = 0.005) with the inclusion of a second blood collection.

### Association between qPCR assay and blood culture in chronic Chagas disease patients

Of the 135 screened samples, 38.5% (52⁄135) were tested positive for both qPCR and BC, 20.0% (27/135) were only positive for qPCR, 11.1% (15/135) were positive for BC but qPCR negative, and 30.4% (41⁄135) were negative for both assays (Table 4).

**Table 4:** Percentage of agreement, point and interval estimates of kappa coefficient of 135 blood samples obtained from 91 Chagas disease patients from different endemic regions of the state of Minas Gerais (southern Brazil).

qPCR	Blood culture	Percentage (Number of patients/total)	Agreement Coefficient	Type of agreement
Positive	Positive	38.5 (52/135)	0.374 [0.205; 0.542]	Fair
Negative	Positive	11.1 (15/135)		
Positive	Negative	20.0 (27/135)		
Negative	Negative	30.4 (41/135)		

**Fig 4:**
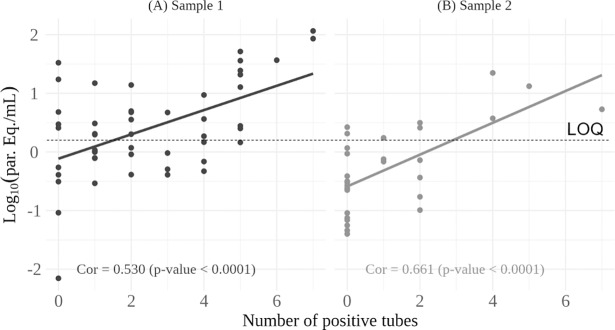
Correlation between parasite load and number of blood culture positive tubes in patients with chronic Chagas disease. The number of positive qPCR results for the first and second clinical samples was respectively, 49 **(A)** and 30 **(B)**. LOQ: Limit of Quantification [25].

The parasitemia of Chagas disease patients was also evaluated by the number of positive tubes for BC and comparing with the parasite load obtained in qPCR of all positive clinical samples. Fig 4 shows a significant correlation between the number of positive tubes in BC and the parasite load of *T*. *cruzi* in clinical samples (p-value<0.0001). We also observed significant correlation between the number of BC positive tubes and the parasite load in the individual analysis of the first and second samples in patients with two blood harvesting (p-value<0.0001) (Fig 4A and 4B).

## Discussion

Due to the sub-patent and transient parasitemia, the direct detection of *T*. *cruzi* in the chronic phase of Chagas disease requires biological amplification methods such as blood culture and xenodiagnosis. These methods are more complex, expensive, time-consuming and require special biosecurity conditions in the laboratory [46,47]. Previous reports have shown that multiplex real-time qPCR assay allowed detection and quantification of parasite DNA from clinical samples with variable levels of reliability, complexity, selectivity and analytical sensitivity [21,23–25,28,31,34], also permitting the *T*. *cruzi* genotyping in clinical samples [33, 48].

In this study, blood samples from chronic Chagas disease patients with well-defined clinical forms were evaluated, using blood culture (BC) and multiplex quantitative real-time PCR (qPCR) to the detection and quantification of *T*. *cruzi* DNA in human blood. All patients presented positive conventional serology for *T*. *cruzi* and had not received any specific etiological treatment. For 44 patients, two blood samples were collected, in an interval of two to three years, in order to evaluate the parasite load of chronic patients in the period of 2011–2014.

Herein, BC was positive in 49.6% (67⁄135) of the clinical samples. In a previous study from our group, we detected 54.9% (50/91) of positive BCs, corresponding to first samples collected from the 91 patients [49]. The vast majority of data in the literature have reported BC positivity ranging from 40 to 70% [6,15,41,49–52]. In the patients with two blood samples, blood culture positivity rate was the same (38.6%) for the first and second blood samples. However, the degree of agreement between the two samples was fair, indicating that a patient with positive BC in the first sample can present positive or negative BC in the analysis of the second blood sample. To the patient 0054, for example, it was observed a positive blood culture at the first sample (0054a) but negative at the second (0054b). Our results confirm previous findings and indicate that at least two blood samples should be collected from chronic Chagas disease patients in order to detect circulating *T*. *cruzi* [15,16,41,53].

The qPCR method has shown higher potential to diagnose and estimate the parasite load, despite the subpatent and transient parasitemia that occurs in the chronic phase of Chagas disease. According to this methodology, the Limit of quantification was reported as 1.53 parasite equivalents/mL [25], which means that samples with parasite load below this limit can be considered detectable but not quantifiable. Nevertheless, as the majority of samples of patients from Brazil are in this condition, we decided to report the parasite load for all the positive samples. Thus, *T*. *cruzi* DNA was detected in 58.5% (79⁄153) of blood samples by qPCR and the median parasite load was 1.18 [0.39–4.23] par. eq.⁄mL, varying between 0.01 and 116.10 par. eq.⁄mL. On the other hand, *T*. *cruzi* k-DNA was detected by conventional PCR in 98.9% (90/91) of the first samples collected from these patients [49], demonstrating more efficiency in detecting the parasite in the peripheral blood of infected patients when compared to BC and qPCR. However, conventional PCR does not allow monitoring parasite load in peripheral blood of chronic Chagas disease patients and as a criteria for the isolation of *T*. *cruzi*, emphasizing the importance of BC and qPCR for new biological, molecular, biochemical, immunological, genetic studies of parasitic populations and parasite load monitoring.

Our findings corroborate with other studies using qPCR to infer parasite load from blood of Brazilian, Argentines, Bolivians, Colombians and Mexicans chronic Chagas disease patients, where the median parasite load ranged from 1.23 to 4.0 par. eq./mL[25,27,28,31, 34]. Data with chronic Chilean Chagas disease patients have demonstrated parasite loads from <0.1 to 78 par. eq.⁄mL [33], higher than previously described for the same group of patients, fluctuating between <0.1 and 7.9 par. eq.⁄mL [30]. Studies have shown that qPCR has been used for the detection and quantification of *T*. *cruzi* load in blood, serum, heart tissue, cord blood, fecal samples of *Triatoma infestans* (xenodiagnosis), skin tissue samples from Chagas disease patients in the acute and chronic phases and also to differentiate the parasite DTUs [21–34,48], suggesting that genetic differences between parasite strains can influence the parasitic load and PCR positivity [20,22,26,27].

In this study, the detection rate for *T*. *cruzi* by qPCR increased from 40.9% to 68.2% in patients with two blood samples collected at different time points; however, the concordance analysis indicated a slight correlation between the samples, with qPCR results from the first sample not often matching the results observed in the second sample.

We performed a comparative analysis of qPCR positivity and blood culture. The two techniques were positive in 38.5% of the samples. Discordant results were observed in 31.1% of the samples, being 11.1% of them positive by BC and qPCR negative, while in 20.0% only the qPCR gave positive results. In contrast, 30.4% of the samples were negative by both techniques. This finding confirms the occurrence of intermittent parasite levels and depends on the number of circulating parasite at the time of blood collection and the number of samples analyzed from the same patient, since in the life cycle of *T*. *cruzi*, the release of trypomastigote forms does not occur in a synchronized way. So, the presence of the parasite in peripheral blood at a given time depends on the parasite’s biological cycle, as well as on the immunological equilibrium among parasite and host [54]. Differences in positivity between qPCR and BC can be explained by low parasitemia, probably below the detection limit of the two techniques.

Understanding the structure of *T*. *cruzi* population is essential due to the links between parasite transmission cycles and the infection⁄disease. *T*. *cruzi* isolates were analyzed by rDNA, COII and SL-IR molecular markers aimed at detecting the six DTUs of *T*. *cruzi*. Most isolates from the patients were associated with DTU II. Two isolates from patients with cardiac and indeterminate clinical form, respectively, were also identified associated with DTU III or IV and DTU V or VI [49]. These data were consistent with previous studies showing that DTU II was associated with human infection in the state of Minas Gerais, Brazil [16,42,55].

Consistent with previous studies, we did not find a correlation between neither *T*. *cruzi* parasite load nor age and clinical presentation of Chagas disease [21,24,26,27,31,33]. This lack of correlation was also observed in another Brazilian cohort comprising 40 patients with chronic Chagas disease [27]. In our recent study, we did not observe significant difference between BC results, age of patients and clinical form [49]. We believe that the lack of association between *T*. *cruzi* parasite load and forms of the disease might be related to parasite tropism for specific organs or host tissues. After their penetration into human tissues, some *T*. *cruzi* populations could disappear, while others could invade different tissues, which would be responsible for the various clinical manifestations in Chagas disease. Thus, parasite obtained in the peripheral blood may not represent the populations of *T*. *cruzi* present in other tissues and/or organs of the patients. Furthermore, it is more important to analyze parasite present in the bloodstream at different time periods, increasing the chance of recovery of different *T*. *cruzi* subpopulations and making possible the analysis of its importance in the pathogenesis of Chagas disease [56,57].

Finally, our results showed a positive correlation between *T*. *cruzi* parasite load estimated by qPCR and number of positive BC tubes, demonstrating a high potential of qPCR for diagnosis and monitoring parasite load in peripheral blood of chronic Chagas disease patients. In another work, the parasitic loads of 15 GEB samples from Brazilian chagasic patients were compared with hemoculture. Despite the small number of samples, these authors demonstrated a good correlation between the parasitic load of *T*. *cruzi* detected by qPCR and the positivity of blood culture. [28].

Our results suggest that qPCR has diagnostic advantages for *T*. *cruzi* detection compared to BC, as it requires low blood volume and shorter processing time, allowing analysis of several samples at the same time. In addition, this tool presents high sensitivity for *T*. *cruzi* detection and quantification with lower risk of sample contamination when compared to BC. Another advantage in the use of the multiplex TaqMan assay is the possibility of checking the quality of patients’ blood processing and DNA extraction, especially to avoid false negative results [21,24–27,31,34]. On the other hand, BC has been frequently used for the isolation of *T*. *cruzi*, a necessary procedure for studies on biological, biochemical, immunological and some genetic aspects of parasite populations. Thus, BC is the most efficient technique for *T*. *cruzi* isolation and its amplification using LIT culture medium [15,16,41].

Taken together, our data suggest that qPCR can be an auxiliary tool for studies that require the isolation of *T*. *cruzi* parasite from the bloodstream of chronic Chagas disease patients, after establishing a cut-off for parasite load assuring a relative success rate for their isolation using blood culture technique.
